# An olive parentage atlas: founder cultivars, regional diversification, and implications for breeding programs

**DOI:** 10.1186/s12870-026-08504-y

**Published:** 2026-03-11

**Authors:** F. Gómez-Gálvez, R. de la Rosa-Navarro, G. Besnard, I. J. Lorite, A. Belaj

**Affiliations:** 1 Instituto Andaluz de Investigación y Formación Agraria, Pesquera, Alimentaria y de la Producción Ecológica (IFAPA), Centro Alameda del Obispo, Avda. Menéndez Pidal s/n, Córdoba, 14004 Spain; 2 Institute for Sustainable Agriculture, Spanish National Research Council (IAS-CSIC), Campus Alameda del Opbispo, Avda. Menéndez Pidal s/n, Córdoba, 14004 Spain; 3 Centre de Recherche sur la Biodiversité et l’Environnement (CRBE), UMR 5300 CNRS-IRD-TINP-UT3, Université Toulouse III – Paul Sabatier, Toulouse cedex 9, 31062 France

**Keywords:** Pedigree atlas, Genealogy, Breeding, Cultivar diversification, Inter-compatibility, Founder cultivars

## Abstract

**Background:**

Olive pedigree has been scarcely explored beyond domestication and diversification studies, even though it can be valuable for breeding programs and germplasm management. This study presents a new and comprehensive exploration of olive parentage relationships by combining a large dataset of 840 cultivars with a cost-effective and highly informative panel of 96 EST-SNP markers routinely applied at the World Olive Germplasm Bank of Córdoba. Parentage assignments were performed combining two approaches, SambaR and CERVUS, and using 13 seedlings of known crosses as validation controls.

**Results:**

This strategy revealed 1,218 parent-offspring duos and 280 robust parent-parent-offspring trios, involving more than 85% of the individuals analysed. Four founder cultivars, ‘Gordal Sevillana’, ‘Lechín de Granada’, ‘Toffehi Tataouine’, and ‘Safrawi’, emerged as central nodes in the pedigree network, highlighting their crucial role in the diversification of olive cultivars across the Mediterranean Basin. ‘Gordal Sevillana’, in combination with ‘Lechín de Granada’ and ‘Toffehi Tataouine’, contributed substantially to the origin of Western and, to a lesser extent, Central Mediterranean cultivars, while ‘Safrawi’ acted as a key connector across the entire basin.

**Conclusions:**

This study provides the first olive parentage atlas, providing new insights into the diversification processes, but also a practical tool for the management of genetic resources. In particular, these results demonstrate that a small but informative SNP set can generate reliable pedigree information to identify compatible parents, resolve uncertain genealogies of cultivars of agronomic interest, reconstruct unknown pedigrees in open-pollination, and guide the selection of balanced parental sets for developing new cultivars.

**Supplementary Information:**

The online version contains supplementary material available at 10.1186/s12870-026-08504-y.

## Background

The olive tree (*Olea europaea* L. subsp. *europaea* var. *europaea*) is one of the earliest woody crops [1, 2]. With an annual worldwide production of 23 million tons of olives produced across 11 million hectares, it represents an important source of local livelihood, mainly in the Mediterranean Basin [3]. Despite its economic and cultural relevance, the genetic background of olive cultivars remains only partially understood.

Exploitation of its wild relative [*Olea europaea* subsp. *europaea* var. *sylvestris* (Mill.) Lehr] is estimated to have occurred since the Neolithic in different parts of the Mediterranean Basin [1, 2]. The domestication of this crop took place ~ 6000–7000 years ago in the Fertile Crescent region of the Middle East, according to evidence from archaeological, palaeobotanical, and genetic studies [1, 2, 4, 5]. As with other cultivated woody species, farmers should have first learned to propagate the most promising individuals by transplanting hard-cuttings and later by grafting [1, 6]. Thus, early olive farmers might have selected the best individuals from wild olive populations according to their fruit size, oil content, productivity, and adaptation to the local environment, giving rise to the first cultivars [1, 5, 7]. Reproduction from seeds may also have occurred, especially during the early stages of domestication, either unintentionally through seed dispersal by migrating people or intentionally promoted by farmers as a means to generate new genetic diversity, as it has been described in other clonally propagated crops [8].

The expansion of the olive crop over centuries, combining both vegetative and sexual propagation, has resulted in several thousand cultivars. Various multilocus genetic analyses have revealed geographical structuring of olive diversity in Eastern, Central, and Western Mediterranean gene pools [5, 9, 10]. However, the modern oliviculture motivates the extensive use of few elite cultivars, which poses a risk of genetic erosion and a decrease in the variability of the crop [11]. This loss of diversity could affect the ability to respond to various challenges of the future, such as climate change, so a comprehensive effort has been put over decades to safeguard, characterize and valorise olive genetic resources in *ex situ* germplasm collections in Mediterranean countries and beyond [12].

Accurately identified and agronomically evaluated *ex situ* germplasm collections can serve as a library that feeds breeding programs in search of new cultivars adapted to the evolving challenges of olive cultivation. In this sense, documenting the history of selected individuals, including their parentage relationships, is a prerequisite for managing germplasm collections and material used in breeding or research. In recent times, molecular markers such as microsatellite and Single Nucleotide Polymorphism (SNP) markers have been used to explore this purpose [13, 14]. Thus, pedigrees have been reconstructed in a wide range of clonally propagated crops, including almond, apple, grape, and peach [15–20]. In the case of olive, few studies have incorporated parentage relationships in their analysis [21–24]. However, these studies were conducted with a contrasting and limited number of cultivars, heterogeneous marker sets, and/or a primary focus on uncovering aspects of domestication history and diversification. As a result, the genealogical links among cultivars of agronomic relevance and the contribution of key founders remain insufficiently assessed.

Beyond reconstructing domestication and diversification events, pedigree analysis can be useful for other purposes in the framework of breeding and genetic management of material in research. First, it may assist for deducing mating behaviours and variations in reproductive success [25]. In this sense, the study of parent-offspring relationships can give important information about the cross compatibility of olive cultivars as olive possesses a self-incompatibility (SI) system characterized by two groups G1/G2: individuals belonging to one group are only compatible with individuals of the other group and vice versa [26–29]. In the same way, the availability of prior information on cross-compatibility groups, together with additional biological information such as the maternally inherited cytoplasmic haplotypes and male sterility, can serve as robustness filters to evaluate and validate inferred parentage relationships. Second, it can help to elucidate an unknown parent in open-pollination crosses. Knowledge of parentage enables identification of successful genetic combinations, facilitates selection for desirable traits, and improves understanding of how variability is transmitted to offspring [30]. Third, the selection of appropriate starting material in breeding programs can be greatly facilitated by pedigree analyses, for example by considering the parentage of progenitors in order to manage relatedness and genetic diversity. This is particularly relevant in the context of contemporary olive growing which increasingly demands the development of low-vigour cultivars adapted to high-density hedgerow systems [31, 32]. Many of the emerging cultivars developed through both public and private breeding initiatives are primarily or secondarily derived from the same progenitor, the ‘Arbequina’ cultivar. While such breeding strategies that increase homozygosity can be effective for fixing desirable agronomic traits, the repeated use of closely related genetic material may increase genetic uniformity and produce a lack of diversification in olive orchards [31–33]. Finally, pedigree information also enhances the management and use of germplasm banks, for example by guiding the selection of maximally diverse cultivar sets for building core collections, comparative trials, or large-scale phenotyping experiments.

In this context, the present study aims to enlarge the knowledge in parentage relationships in olive and complementing it from a more practical point of view for breeding and management of olive germplasm collections. To achieve this, our study takes advantage of a large dataset of cultivars accurately identified by a set of 96 EST-SNPs routinely applied at the World Olive Germplasm Bank of Córdoba (WOGBC-ESP046) and its associated prospecting trials [10, 11, 34]. It is worth mentioning that the use of a relatively small panel of SNPs genotyped via real-time PCR (Fluidigim) has proven efficacy for parentage analysis in other species [14]. Our study represents the first attempt to do so in olive, demonstrating that informative set of 96 EST-SNPs is enough to routinely perform Germplasm-Bank-scale pedigree analysis within a reasonable timeframe and without the need for high-performance hardware, i.e., in a cost-efficient way. Using this marker set, we conducted paternity assignments for 853 olive individuals, representing, to our knowledge, the largest dataset used for parentage analysis in olive to date. The resulting pedigree relationships provide novel insights into the diversification processes of the crop across the Mediterranean, shed light on mating behaviours among cultivars, and offer valuable guidance for the selection of genitors in future breeding crosses.

## Materials and methods

### Material

All the distinct cultivars identified in previous studies were subjected to parentage analysis in the present work. Thus, 668 different international cultivars, genotypically identified by Belaj et al. [10]. together with 172 different Spanish cultivars, genotypically identified by Gómez-Gálvez et al. [11]. were used. In addition, 13 seedlings were included as validation controls, 12 of them from both known genitors and one from a known mother fertilized in open pollination. To sum up, a total of 853 distinct individuals were analysed (Table S1). All genotypic data were generated using the same standardized genotyping pipeline based on the 96 EST-SNPs panel routinely applied at the WOGBC-ESP046 [10]. DNA extraction, genotyping methodology and allele calling procedures were identical across studies, ensuring full data consistency and comparability. Descriptive statistics, including allele frequency distribution, minor allele frequency (MAF), and missing data rates, were calculated for each SNP. The calculation was made both for the complete dataset of 853 individuals and separately for cultivars grouped by main region of cultivation (Eastern, Central, and Western Mediterranean; EM, CM, and WM, respectively). This regional grouping was based on the passport information available for each cultivar in the WOGBC-ESP046 database, specifically considering the area where each cultivar is predominantly grown [10].

Information on maternal lineages (i.e., chloroplast DNA data), and stigmatic groups of cross-compatibility (G1/G2) was added for each cultivar in Table S1 according to information available in literature (see references in Table S1). Some andro-sterility information obtained from the periodic phenology records routinely carried out in the WOGBC-ESP046 was also added (at the beginning of flowering, andro-sterile cultivars can be detected by observing dehiscence of the anthers and/or absence of pollen). Finally, information of population structure was also presented in Table S1. Since this information was separately explored in previous works for the different sets of cultivars [10, 11], a new exploration was conducted here combining all the cultivars under study. Thus, this new information of population structure was obtained using ‘LEA’ admixture analysis within the pipeline of SambaR package [35] (https://github.com/mennodejong1986/SambaR) performed in R v.4.2.2 [36]. The *snmf* (*sparse nonnegative matrix factorization*) algorithm was run, and the optimal number of populations (*K*) was assessed based on the cross-entropy criterion through examining a range of *K* from 1 to 10 with 10 replicates for each scenario [37]. The proportions of membership (Q) of each individual in each cluster were exported. A cultivar was considered belonging to a cluster when its Q > 75%; otherwise, it was considered as admixed.

### Parent-offspring analysis (duos)

Parentage analysis was conducted following two steps, similar to the procedure published by Khadari et al. [22]. First, a single-parent search was performed in the whole set of 853 distinct individuals to identify putative parent-offspring duos. Second, a parental pair search was conducted on the individuals inferred in some putative parent-offspring duos to identify putative parent-parent-offspring trios. For parent-offspring duos, two different approaches were used: (i) First, the kinship analysis available in the R package SambaR was used. This analysis does not require allele frequency information and is based on the approach of Manichaikul et al. [38]. Thus, a parent-offspring duo was retained if all SNP loci shared at least one allele (i.e., the “zero identity-by-state”, R0, coefficient is zero) and if the KING-robust coefficient (φ) was within the inference criteria range (0.177 < φ < 0.354) [38]; (ii) Second, the parentage analysis available in CERVUS v.3.0.7 was computed [39]. This analysis relies on allele frequencies and is based on the difference in the log-likelihood ratio (LOD) between related and unrelated relationships to assign parentage combined with simulation of parentage analysis to determine the confidence of assignments [40]. The default settings for the simulation, recommended by the software [39], were the following: number of offspring = 10,000; number of candidate parents = 200; proportion of candidate parents sampled = 0.3; proportion of loci typed = 0.8; and proportion of loci mistyped = 0.01. Two criteria were finally considered to establish strict parent-offspring relationships: a confidence level of the duo LOD score (LOD_p_) higher than 95% and mismatches on a maximum of two loci. Such parameters have also been used in previous works on woody crops [22, 24, 41, 42].

### Parent-parent-offspring analysis (trios)

For parent-parent-offspring trios, all those cultivars inferred in any duo were included in the likelihood-based method of CERVUS v.3.0.7 using the same parameters and criteria as above [39]. From the putative trios obtained, a selection of the most robust ones for each offspring was done and represented in a pedigree network.

Robust trios selection was done according to the higher trio LOD score (LOD_pp_). In cases where multiple parental pairs with similar LOD_pp_ values were inferred for the same offspring, trios were further evaluated by prioritizing (i) the lowest number of trio mismatching loci, (ii) the number of parents significantly supported in the previous duo analyses, and (iii) consistency with known geographic distribution. In cases where a high number (> 20) of statistically supported parental pairs were inferred for a single offspring, no trio was selected, as this pattern was interpreted as the offspring acting predominantly as a candidate parent rather than representing a true descendant.

Ambiguous cases in which the same cultivars appeared in multiple trios, either as offspring or as parents, were resolved by retaining the trio with the highest LOD_pp_ value.

In addition, when available, additional information (chloroplast haplotypes, stigmatic compatibility groups, and andro-sterility) was used as additional filter of validation.

### Sensitivity analysis of trio inference

Additional sensitivity analyses were performed in CERVUS v.3.0.7 to evaluate the robustness of parent–parent–offspring trio inference, i.e., to assess the behaviour of the method when one or both true parents are absent from the reference dataset. These analyses were conducted on the 13 validation seedlings assuming different sampling scenarios in the candidate parent set. Thus, in addition to the baseline analysis using the complete reference dataset (scenario 1), trio inference was repeated after removing a known parent of each validation offspring from the candidate parent set (scenario 2), and after removing both known parents (scenario 3). All analyses were conducted using the same inference framework and thresholds as the baseline analysis. Trio support was evaluated using the number of loci compared, the number of mismatching loci, and the LOD_pp_ and confidence values.

### Use of additional information in robust trios

Information of chloroplast data, stigmatic groups of compatibility, and andro-sterility (see reference sources in Table S1) was incorporated to all the robust trios selected. This information was first used to corroborate the selection of robust trios, i.e., checking the plausibility of the matching when the information of the trio was complete. Chlorotype and andro-sterility information were also used to deduce the sex of candidate parents when possible. Finally, the stigmatic information was used to deduce the compatibility groups in those robust trios with partial information, i.e., if a candidate parent with an unknown group was inferred as a pair of a candidate parent with stigmatic group G1, it was deduced as G2, and vice versa.

## Results

### Descriptive statistics of markers

Descriptive statistics of the 96 EST-SNPs are summarized in Table S2. All loci showed a high genotyping success, with an average of 850.6 genotyped individuals per locus, corresponding to a mean missing data rate of 0.28%. The SNP panel displayed intermediate to high levels of polymorphism, with a mean expected heterozygosity (*H*_*e*_) of 0.46 and a mean polymorphic information content (PIC) of 0.35. Minor allele frequencies (MAF) were generally intermediate across loci, with a mean MAF of 0.38, indicating a high proportion of informative markers. Comparable levels of polymorphism were observed when markers were evaluated separately in the Eastern, Central, and Western Mediterranean groups, indicating a consistent segregation of alleles across populations.

### Genetic structure

The ‘LEA’ admixture analysis detected *K* = 3 as the most likely number of genetic groups, so three clusters were considered, denoted as cluster groups A, B, and C. The proportions of membership (Q) of each individual in each cluster are presented in Table S1 and Figure S1. For Q > 75%, 145 cultivars were considered belonging to cluster A, 51 to B, and 163 to C. The rest of cultivars (494; 58%) were considered to be admixed (Q < 75% in any cluster). Most of cultivars assigned to clusters A, B, and C belong to cultivars mainly grown in Eastern, Central, and Western Mediterranean countries, respectively (Table 1; Table S1; Figure S1).

**Table 1:** Averaged proportion of membership (Q) observed for cultivars grown in Eastern (EM), Central (CM), or Western (WM) Mediterranean countries and number of cultivars (N) assigned to each cluster (Q > 75%) or admixed (Q < 75%)

Region of cultivation	Q mean for A	Q mean for B	Q mean for C	*N* assigned to A	*N* assigned to B	*N* assigned to C	*N* assigned as admixed
EM	0.764	0.089	0.147	97	0	1	56
CM	0.345	0.406	0.249	19	39	2	153
WM	0.283	0.190	0.527	29	12	160	285

### Parent-offspring duo analysis

A total of 1,696 different putative parent-offspring duos were identified in SambaR. With this method, around 89% (759 out of 853) of the individuals under study conformed at least one duo (Table S3). The CERVUS approach identified 3,221 different putative parent-offspring with 95% confidence level (LOD_p_ > 4.65) and less than two mismatching loci (Table S3). In this case, 815 cultivars (95.6%) were inferred in at least one duo.

**Fig. 1:**
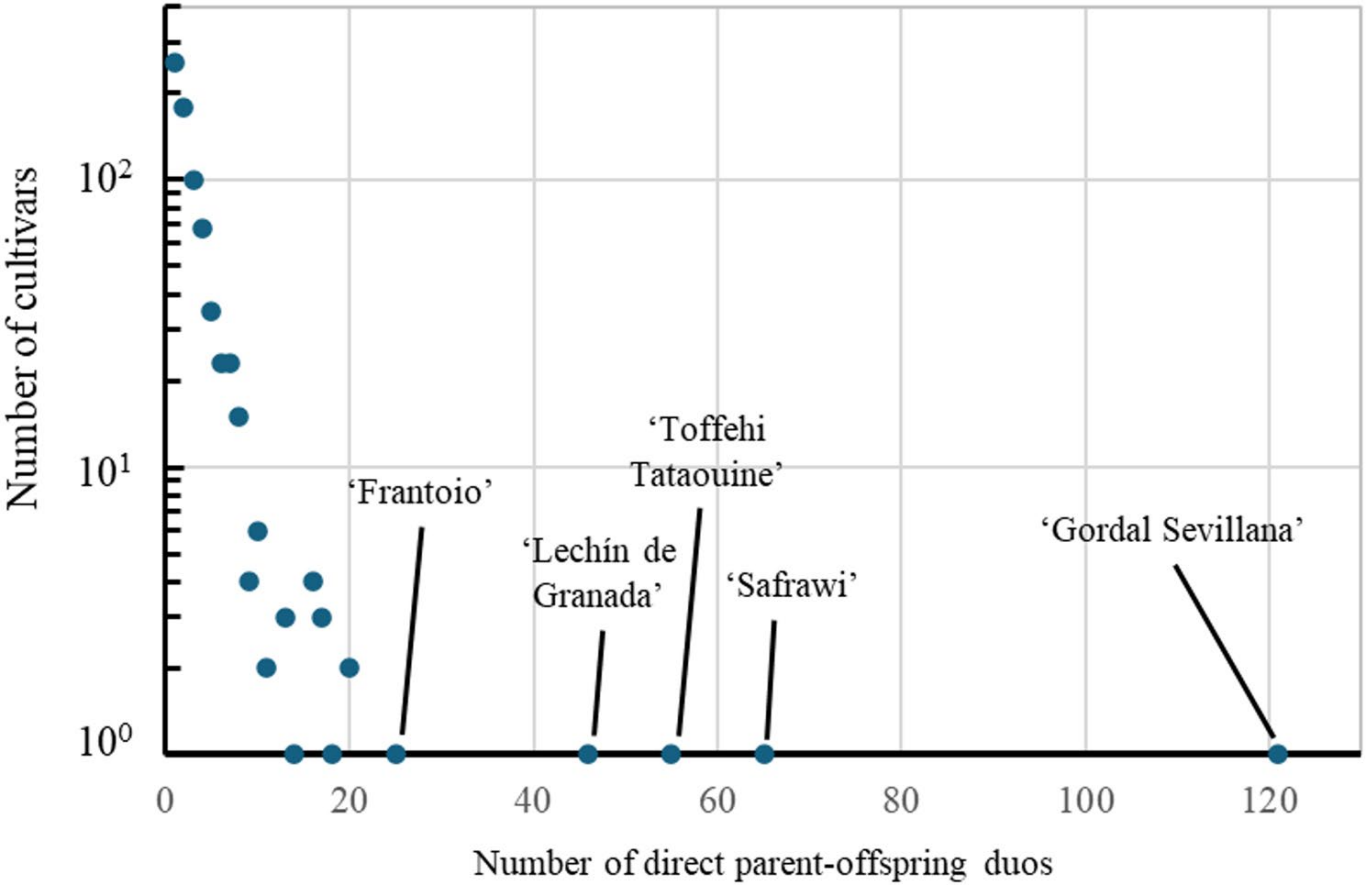
Number of direct parent-offspring duos inferred for each cultivar jointly by SambaR and CERVUS. The names of the five cultivars with more pairwise relationships (> 25) are indicated

The number of putative duos validated by the two approaches was 1,218, and more than 85% of the individuals conformed pairwise relationship(s) (Table S3). Of these, 255 individuals were inferred only once, while 27 were inferred in 10 or more parent-offspring duos. Among the latter, ‘Gordal Sevillana’, ‘Safrawi’, ‘Toffehi Tataouine’, and ‘Lechín de Granada’ showed more than 45 parent-offspring relationships (Fig. 1), pointing out their founding importance in the current olive germplasm. It is worth noting the wide geographical range of relationships for two of them: firstly, ‘Gordal Sevillana’ shows direct relationship with well-known cultivars found in the East (e.g. ‘Izmir Sofralik, ‘Gemlik’, or ‘Sebhawy’), the Centre (e.g. ‘Amygdalolia Nana’, ‘Ascolana Tenera’, or ‘Buga’), and the West (e.g. ‘Azapa’, ‘Bouteillan’, or ‘Manzanilla de Sevilla’); and secondly, ‘Safrawi’ is also related to eastern (e.g. ‘Rowghani’, ‘Tebabs’, and ‘Ayvalik’), central (e.g. ‘Karydolia’, ‘Gerboui’, and ‘Dolce Agogia’), and western cultivars (e.g. ‘Aubenc’, ‘Panseñera’, and ‘Empeltre’; Table S3). In contrast, the three other founder cultivars (i.e., ‘Toffehi Tataouine’, ‘Lechín de Granada’, and ‘Frantoio’) were related to cultivars mainly found in the Central and Western olive growing areas.

**Fig. 2:**
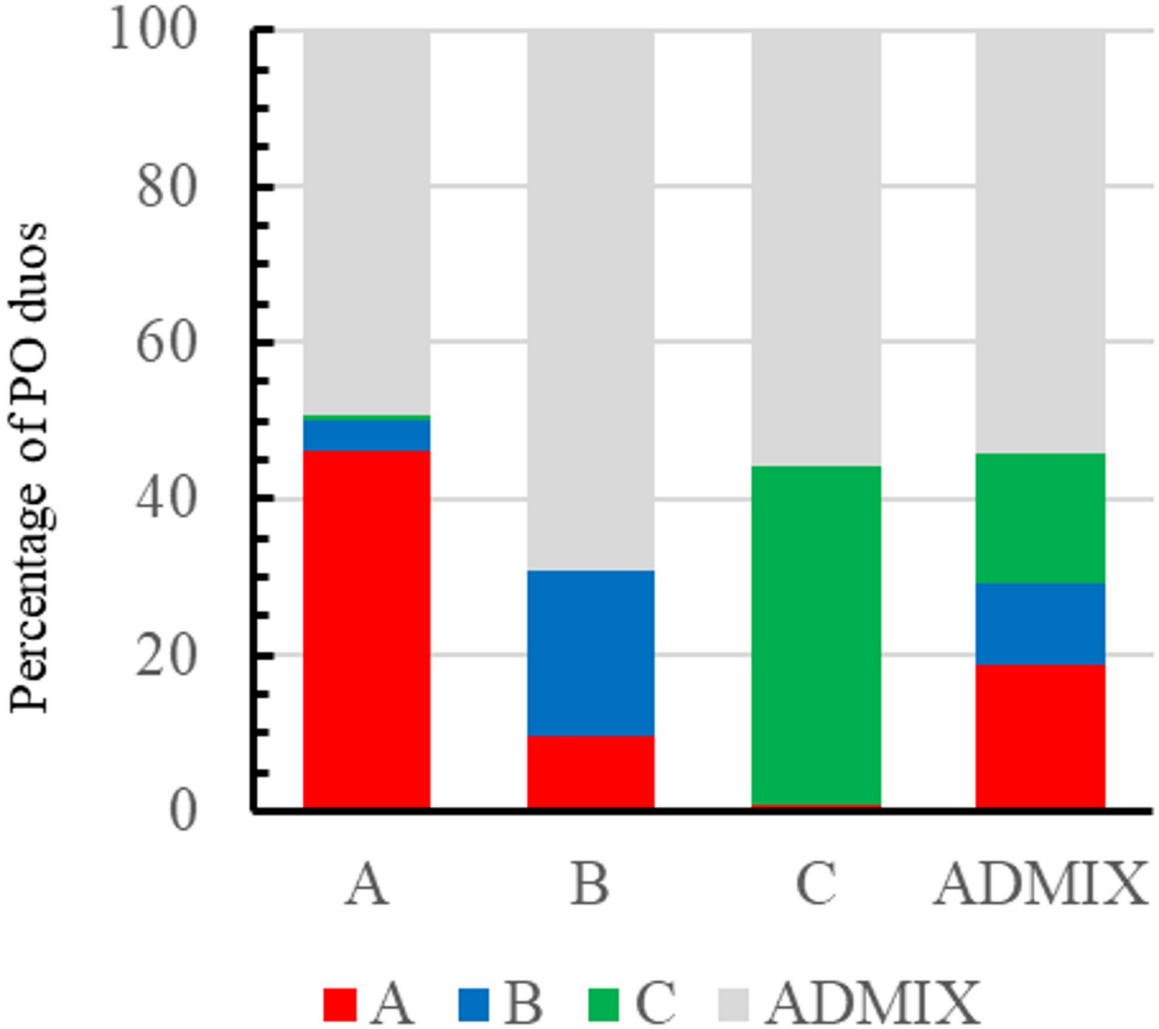
Percentage distribution of parent-offspring (PO) duos according to the cluster assignment in the LEA analysis. The x-axis shows the cluster assignment of the parental cultivars, and each column represents the cluster assignment of the offspring. A (red), B (blue) or C (green) represent the three clusters with membership assignations Q > 0.75. The ADMIX (grey) category represents admixed cultivars with Q < 0.75

Most duos were identified among cultivars belonging to the same cluster or among cultivars belonging to a cluster and admixed ones (Fig. 2). Duos identified for cultivars belonging to cluster A (Eastern Mediterranean) were inferred in 46% of the cases with other cultivars of the same cluster and 49% with admixed cultivars. As for cluster B (Central Mediterranean area), 21% of the duos were identified within the same cluster, and 69% were found among admixed cultivars. Finally, those assigned to cluster C (Western Mediterranean) appeared in duos with cultivars from the same cluster (43%) or with admixed ones (56%). Only 13 duos included a cultivar belonging to cluster A (either ‘Toffehi Tataouine’, or ‘Ayvalik’) and a cultivar belonging to cluster B; only two duos (‘Toffehi Tataouine’ – ‘Verdale de l’Hérault’, and ‘Bassiols’ – ‘Cornicabra’) were conformed among a cultivar belonging to cluster A and cluster C; and none of the duos were inferred among cultivars belonging to B and C clusters (Fig. 2).

### Parent-parent-offspring trio analysis

A total of 1,000 putative trios were identified under the criteria considered, i.e., LOD_pp_ > 14.88 for 95% success rate, maximum two mismatching loci, and both parents inferred as significant in at least one of the approaches conducted in previous duo analysis (Table S4; Figure S2).

From these putative trios, a selection was done to conserve the most likely parental pair for each offspring (according to the higher LOD_pp_ and other considerations; see criteria above). Thus, a total of 142 individuals were involved in 131 different parental pairs leading to 280 different offspring individuals, with LOD_pp_ scores ranging from 15.69 to 58.38 (Table S5; Figure S2).

All validation controls were recovered within these robust trios. In all the cases in which both parents were known, offspring were assigned to the expected parental pairs, with trio LOD_pp_ scores ranging from 19.65 to 37.30. For example, ‘Martina’, ‘Chiquitita’, and ‘Sikitita-2’ were correctly assigned to the expected parental combination ‘Arbequina’ × ‘Picual’, with LOD_pp_ scores of 19.65, 28.37, and 27.27, respectively (Table S5). The robustness of these assignments was further evaluated through additional sensitivity analyses using the validation offspring (Table S6). When one or both known parents were removed from the candidate parent set (scenarios 2 and 3, respectively), trio assignments frequently collapsed or showed markedly reduced statistical support, with lower LOD_pp_ and increased numbers of mismatching loci compared to the baseline analysis (scenario 1). No alternative high-confidence trios, i.e., matching with the robustness criteria, were identified in scenarios 2 and 3, confirming the reliability of the approach.

Among the 280 robustly identified trios, the distribution of offspring cultivars by main region of cultivation was as follows: 51 from the Eastern Mediterranean (EM), 61 from the Central Mediterranean (CM), and 168 from the Western Mediterranean (WM) (Table S5). If combining with the main region of cultivation of parents-pairs, footprints of local selection were revealed, with a clear predominance of within-region crosses. Thus, crosses among WM cultivars led to 119 offspring cultivars in the same region; crosses among CM cultivars gave 31 CM cultivars; and crosses among EM cultivars produced 29 EM cultivars. Nevertheless, a notable number of inter-regional combinations were also observed, including EM–CM (25 offspring cultivars), CM–WM (22), and EM-WM (9). Offspring from the WM exhibited the highest number and diversity of parental combinations, which may be attributed, partially, to their greater representation in the dataset (≈ 57%). In contrast, EM offspring showed more limited parental origins, mostly involving EM or CM cultivars (Table S5).

**Fig. 3:**
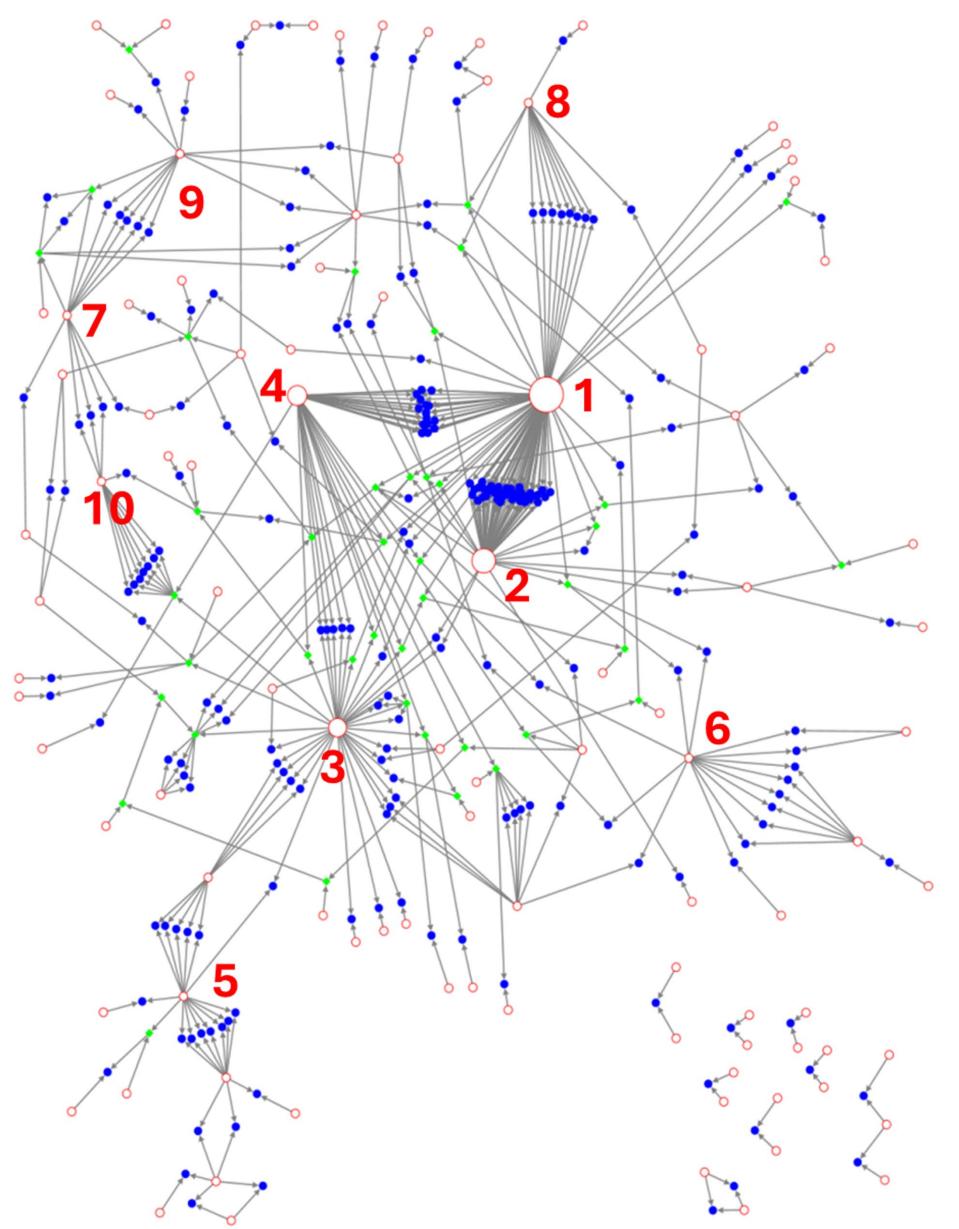
Pedigree network of robust selected trios. Blue spheres = offspring cultivars; Red circles = candidate genitors; Green diamond = offspring and genitors cultivars. Created with NodeXL. Cultivars with more than 10 connections are numbered: 1 = Gordal Sevillana; 2 = Lechín de Granada; 3 = Safrawi; 4 = Toffehi Tataouine; 5 = Zaity; 6 = Chemlali Sfax; 7 = Arbequina; 8 = Morchiaio; 9 = Picual; 10 = Verdal del Pallars

**Fig. 4:**
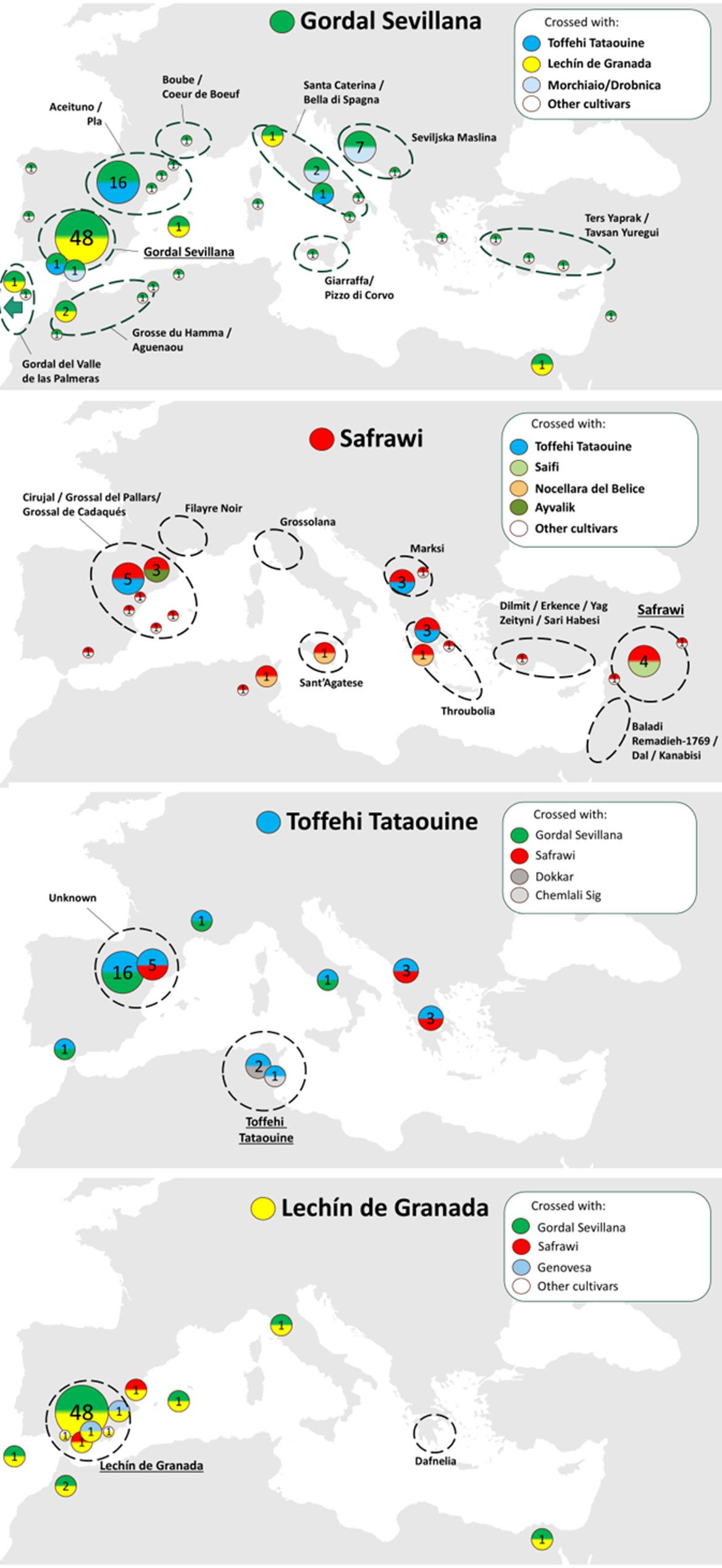
Offspring distribution from the four main founder cultivars. Each circle represents the offspring number derived from parental pairs. Each parent contribution is represented by a colour. Dashed circles represent areas of cultivation of the founder cultivar. The names next to the dashed circles represent synonymies identified in those areas

Representation of the selected robust trios as a pedigree network revealed a main continuous network connecting most genotypes, as well as eight smaller subnetworks composed of a limited number of cultivars (Fig. 3). In the continuous network, several highly connected nodes could be differentiated, representing founder cultivars connections. ‘Gordal Sevillana’, ‘Lechín de Granada’, ‘Toffehi Tataouine’, and ‘Safrawi’ were identified as the most likely candidate parents of 47.5% of the offspring (132 out of 280 cultivars; Figs. 3 and 4; Table S5).

### Contribution of major founders to the Mediterranean olive germplasm

The cultivar ‘Gordal Sevillana’ was inferred as candidate parent in a total of 91 robust trios (Figs. 3 and 4; Table S5). It was inferred in 12 different parental pairs, three of them accounting for most of the offspring: (i) ‘Gordal Sevillana’ × ‘Lechín de Granada’, that was identified as the most likely parental pair for 53 cultivars, mainly cultivated in Southwestern Mediterranean and including important commercial cultivars such as ‘Cornicabra’, ‘Manzanilla de Sevilla’, ‘Cordovil de Serpa’, ‘Picholine Marocaine’, or the South American cultivar ‘Azapa’; (ii) the parental pair ‘Toffehi Tataouine’ × ‘Gordal Sevillana’, reported here for the first time, was directly linked to 19 cultivars mainly cultivated in northern Spain but also in the southern part of the country (‘Amargoso’), in France (‘Grossanne’), and in the Italian island of Sicily (‘Abunara’); and (iii) the pair ‘Gordal Sevillana’ × ‘Morchiaio’, with 10 offspring cultivars, mainly from the Balkan area (7), such as ‘Buga’ and ‘Oblica’, but also including the well-known Italian cultivar ‘Ascolana Tenera’ (Fig. 4). In addition, ‘Gordal Sevillana’ showed close genetic relationships with cultivars from both the Eastern Mediterranean (‘Izmir Sofralik’, ‘Samanli’, ‘Samsun Salamuralik’, and ‘Samsun Tuzlamalik’) and Central Mediterranean (‘Derdi’, ‘Semidana’, and ‘Rotondella di Melfi’). This frequent connection across regions is consistent with its hypothesized eastern origin and later diffusion to the West under different local names, such as ‘Ters Yaprak’ or ‘Tavsan Yuregui’ in Turkey, ‘Seviljska Maslina’ in Croatia, ‘Bella di Spagna’, ‘Giarrafa’, ‘Pizzo di Corvo’ or ‘Santa Caterina’ in Italy, ‘Grosse du Hamma’ in Algeria, or ‘Boube’ in France (Fig. 4). In line with this, chloroplast data further indicate that ‘Gordal Sevillana’ (E1.2 chlorotype) acted as a pollen donor in most of these crosses (offspring generally harbouring the E1.1 chlorotype), reinforcing its role as a male founder (Table S5).

The cultivar ‘Lechín de Granada’ was identified as putative parent of a large group of offspring cultivars (57) in the Southwestern Mediterranean Basin. Most of these cultivars (51) are derived from a cross with ‘Gordal Sevillana’. However, additional parentage combinations were detected in this region, including crosses with ‘Safrawi’ and the local Eastern Spanish cultivar ‘Genovesa’. With the latter, it gave rise to the commercial cultivar ‘Changlot Real’. Beyond this regional influence in the Southwestern Mediterranean Basin, ‘Lechín de Granada’, which is known to be synonymous of the Greek cultivar ‘Dafnelia’, was also identified as a putative parent of the Egyptian cultivar ‘Sebhawy’ and the Italian cultivar ‘Rotondella di Melfi’ (Fig. 4; Table S5).

The founder cultivar ‘Toffehi Tataouine’, introduced in the WOGBC-ESP046 collection from southern Tunisia, and recently discovered on an ancient tree in northern Spain, was inferred in four different parental pairs. Apart from the above-mentioned parental pair with ‘Gordal Sevillana’, its most prolific pairing was with ‘Safrawi’, producing 11 offspring cultivars. These descendants were mainly found across the central and western Mediterranean, including Greek (‘Agouromanakolia’, ‘Kothreiki’), Albanian (‘Kokerramdh Elbasani’, ‘Kotruvsi’), and northern Spanish cultivars (‘Claramunt’, ‘Domengues’). The well-known Turkish cultivar ‘Ayvalik’, synonymy of ‘Edremit Yaglik’ and ‘Adramitini’, was also found to be originated from this cross. Additionally, part of the local Tunisian germplasm may have resulted from crosses between ‘Toffehi Tataouine’ and other national cultivars, such as ‘Chemlali Sig’ and ‘Dokkar’ (Fig. 4).

The cultivar ‘Safrawi’ was inferred as candidate parent in 14 different parental pairs, generating 34 offspring cultivars. It was the most frequently inferred founder cultivar in parental pairs (7) that generated more than one descendant. Thus, besides the above-mentioned pairings with ‘Toffehi Tataouine’ and ‘Lechín de Granada’, it was involved in five additional crosses generating more than one offspring: with Eastern Mediterranean cultivars from Syria (‘Saifi’) or Turkey (‘Ayvalik), as well as with Central Mediterranean ones from Greece (‘Amygdaloia Nana’) or Italy (‘Nocellara del Bellice’ and ‘Ogliarola del Bradano’). It is noteworthy to mention that ‘Safrawi’ was the founder cultivar with the most widely distributed progeny across the Mediterranean area. In addition to local offspring produced in Syria (e.g., ‘Khoukhe’, ‘Sayfi’), it generated other cultivars all along the Mediterranean Basin, such as the cultivars ‘Rowghani’ in Iran, ‘Memeli’ in Turkey, ‘Karydolia’ in Greece, ‘Ulliri shekullor Berat’ in Albania, ‘Aitana’ in Italy, ‘Gerboui’ in Tunisia, and ‘Farga’ in Spain (Fig. 3). This broad distribution and high number of ‘Safrawi’ descendants may probably be due to the extensive dissemination of this variety along the Mediterranean basin where it “was baptised” with different local names such as ‘Antawi’ or ‘Dan’ in Syria, ‘Dal’ or ‘Baladi Remadieh’ in Lebanon, ‘Karabisi’ in Jordan, ‘Celebi (Silifke)’, ‘Erkence’, ‘Hurma Kaba’, ‘Sari Hebsi (Hatay)’, or ‘Yag Zeytini’ in Turkey, ‘Throubolia’ in Greece, ‘Marski’ in Albania, ‘Grossolana’ or ‘Santagatesse’ in Italy, ‘Filayre Noir’ in France, and ‘Cirujal’ or ’Grossal de Pallars’ in Spain (Fig. 4).

Apart from the founder cultivars mentioned above, the network reflected a high number of further connections. They included cultivars that generated offspring in their nearby areas of cultivation, therefore representing local diversification foci. Thus, in the Eastern Mediterranean, the parental pair ‘Zaity’ × ‘Saifi’ originated several cultivars in Syria, while ‘Zaity’ × ‘Baladi’, from neighbouring Lebanon, generated at least seven local descendants. Similarly, six cultivars grown in Egypt were inferred as descendant from the parental pair ‘Chemlali Sfax’ × ‘Balady’, from Tunisia and Egypt, respectively (Table S5). In Central Mediterranean, it was observed local diversity derived from second generation of crosses, i.e., some inferred offspring cultivars were also detected as putative parents of other cultivars. Thus, the Sicilian cultivar ‘Abunara’ (inferred as offspring of the parental pair ‘Toffehi Tataouine‘ × ‘Gordal Sevillana’) was found to be candidate parent of the well-known Tunisian cultivar ‘Meski’. Similarly, four local cultivars in this country seem to have been derived from crosses of the cultivar ‘Jemri Bouchouka’ (a local offspring of the parental pair ‘Toffehi Tataouine’ × ‘Chemlali Sig’) with the Sicilian cultivar ‘Nocellara del Belice’ that was also found in Tunisia with different names (‘Beldi’, ‘Fakhari’, ‘Meski Zarzis’, and ‘Zarrazi’) [10]. This was also found in Western Mediterranean, where, as an example, the cultivar ‘Claramunt’, derived from the parental pair ‘Toffehi Tataouine’ × ‘Safrawi’, gave rise to new local germplasm by pairing with the local cultivar ‘Verdal de Pallars’. Similarly, second-generation crosses seem to have contributed to the local diversity of olive cultivars in the Americas, as some cultivars identified in South America, such as ‘Carrasqueña Huasco’ and ‘Ascolana Huasco’, appear to be descendants of ‘Azapa’, which, as mentioned above, is derived from the parental pair ‘Lechín de Granada’ × ‘Gordal Sevillana’.

### Valuable information deduced from robust trios

Complete information on chloroplast haplotypes, stigmatic compatibility groups, and andro-sterility was available for some of the inferred robust trios. In all these cases, no inconsistencies were detected between the information and the inferred parentage relationships.

When such information was incomplete or unavailable, it could be predicted by means logical deductions based on pedigree structure. This deduced information was consistent with the parentage relationships, even for trios lacking direct bibliographic support (Table S5).

The logical deductions allowed to predict the stigmatic group of 74 cultivars, including cultivars of major agronomic relevance such as ‘Chemlali Sfax’ (G1), ‘Galega Vulgar’ (G1), ‘Azapa’ (G2), ‘Zaity’ (G1), or the founder cultivar ‘Toffehi Tataouine’ (G2). Likewise, the analysis revealed new insights into the reproductive compatibilities of cultivars with observed potential for hedgerow systems, such as ‘Arróniz’, ‘Rotondella di Melfi’, or ‘Zeitoun Boubezzoula’.

Also, the robust trios obtained enabled to deduce the chlorotype information in 52 cultivars and the sex of candidate parents in 60 trios. Most of the chlorotypes deduced (46) were E1.1, as a result of obtaining an offspring with lacking information but originated from a parental pair with both candidate parents classified as E1.1. The combination with andro-sterility information enabled to deduce the chlorotype on a group of cultivars from eastern Spain, for which both types of information were consistent and mutually reinforcing. In particular, *‘*Carrasco’, ‘Cuquello de la Jana’, ‘Picuda de Luis’, and ‘Rufina’ were found to share the same chlorotype (E3.1) and andro-sterility profile, both likely inherited from ‘Farga’, their inferred maternal parent.

Finally, the information provided by the robust trios made possible to identify the male parent in progenies derived from open-pollination in some breeding programs. As an example, the cultivar ‘Favolosa’, obtained in a breeding program from ‘Frantoio’ in open pollination, was identified as offspring of ‘Frantoio’ and ‘Ascolana Tenera’ with a high LOD_pp_ score (43.32). In addition, the approach allowed for the reconstruction of complete pedigrees for cultivars whose origin was only partially known. Notable examples include ‘Dwarf D’, ‘Cairo-7’, or ‘S:27_4’. Similarly, ‘Arbosana’, a commercial cultivar adapted to hedgerow system, was correctly inferred to its known female parent, ‘Arbequina’, and the initially unknown male parent was newly postulated as ‘Vaneta’, a local cultivar that is present in the same area of cultivation.

## Discussion

This present study provides the most comprehensive analysis to date of parentage relationships in olive, revealing both parent-offspring duos and parent-parent-offspring trios within a set of 853 individuals. This large-scale approach, based on a cost-effective set of 96 EST-SNPs, uncovered four main founder cultivars, namely ‘Gordal Sevillana’, ‘Lechín de Granada’, ‘Toffehi Tataouine’, and ‘Safrawi’, that played central roles in the diversification of the crop. Moreover, the resulting pedigree atlas sheds new light on the genealogical structure of Mediterranean olive germplasm and provided practical tools for breeding and germplasm management, moving beyond previous studies that focused mainly on domestication history and diversification.

### Founder cultivars shaping Mediterranean olive diversification

As in other woody plants, analysing parentage is a useful approach to trace the origins of cultivated germplasm. The few generations since domestication and the vegetative propagation of key cultivars may help to reconstruct genealogies and identify founder individuals [19, 44]. Here, various cultivars have been identified as founder cultivars, as evidenced by the large number of the offspring generated. Three of them, ‘Gordal Sevillana’, ‘Lechín de Granada’, and ‘Safrawi’ have been already distinguished as founder cultivars in previous works [21, 22, 24], but here their offspring relationships were expanded and refined thanks to the broader dataset of 853 individuals analysed. In addition, we provide the first evidence about ‘Toffehi Tataouine’ acting as a founder, with descendants in both Central and Western Mediterranean. This represents a surprising finding, as the impact of this cultivar, primarily discovered in Tunisia, has not been suggested until now. Its previously unrecognized influence highlights the importance of including ancient, forgotten cultivars in pedigree studies. Interestingly, an ancient olive tree recently genotyped in northern Spain matched with this cultivar [43], reinforcing the results obtained in our study and supporting for its historical diffusion beyond Tunisia.

A common feature of the four founder cultivars is their association with very old olive trees [9, 24], which reinforces their antiquity and probable early selection. Moreover, ‘Gordal Sevillana’, ‘Lechín de Granada’, and ‘Safrawi’ present well-known synonymies in different parts of the Mediterranean area [10, 22, 24], indicating long-distance dissemination and local renaming that contributed to diversification in different areas. Although ‘Toffehi Tataouine’ currently remains a little-known local cultivar in southern Tunisia [45], the recent evidence of its genetic match in Spain [43] and its prominent role in the inferred offspring suggest that it may also have experienced wider historical diffusion, and future prospecting may reveal additional synonymies. Overall, the identification of these four founders, especially their wide geographical spread and contribution to cultivar diversification highlight the long-distance dispersal and hybridization events that shaped the current structure of Mediterranean olive germplasm. The resulting olive pedigree atlas provides, for the first time, an integrated view of these genealogical links, revealing patterns of crossing between local and foreign cultivars and identifying multiple foci of diversification across the Mediterranean Basin.

### Regional patterns of diversification: Eastern, Central, and Western Mediterranean

In the Eastern Mediterranean region, considered the primary centre of olive domestication, our parentage analysis revealed a remarkable number of cultivars involved in different duos and trios, confirming the important role of eastern cultivars in the early diversification of the crop. Despite the relatively limited number of cultivars from this area included in our dataset (≈ 18%), the results highlight their contribution to multiple, locally adapted hybridization events. This observation is consistent with the high levels of genetic diversity previously reported for the region and with archaeological and genetic evidences indicating that the Levant is the cradle of olive cultivation [1, 4, 5, 24].

In the Central Mediterranean, the parentage network reveals a more complex and heterogeneous pattern of cultivar origin and diversification. This area appears as a transitional zone where both autochthonous and allochthonous individuals contributed to shaping the current olive germplasm. This pattern has been particularly observed in studies conducted in some islands such as Malta, Sicily, or Capri, considered crossroads of the Mediterranean [46–48].

In the Western Mediterranean, the parentage patterns showed a strong founder effect driven by a limited number of recurrent parental pairs. The allochthonous origin of most cultivars of the Western Mediterranean has been previously postulated [5, 21, 22, 24]. In this study, it has been corroborated that the parental pair ‘Lechín de Granada’ × ‘Gordal Sevillana’ had a crucial role in this area, generating a large number of offspring cultivars. Among them, some popular and relevant cultivars such as ‘Cordovil de Serpa’, ‘Manzanilla de Sevilla’, or ‘Picholine Marocaine’, were robustly inferred. Additionally, other commercially important Spanish cultivars, namely ‘Picual’ and ‘Hojiblanca’, were also inferred as descendant of this parental pair, although their trio assignments did not reach the criteria required under our robustness thresholds (data not shown). These genealogical connections should be re-evaluated and confirmed in future analyses using higher-density SNP arrays or whole-genome sequencing.

In the Americas, as in the Central and Western Mediterranean, second-generation crosses appear to have contributed to the local diversification of olive cultivars. For instance, the South American variety ‘Azapa’, identified as an offspring of the parental pair ‘Lechín de Granada’ × ‘Gordal Sevillana’ and so likely introduced from the Iberian Peninsula, was found to act as a progenitor of several local cultivars in that region. Thus, throughout the history of olive cultivation in the Americas, both the introduction and preservation of ancient Mediterranean cultivars—some of which may have been lost or remain to be identified in their regions of origin—together with the local selection and further propagation of seedlings, may have shaped the current olive diversity observed in that area [10].

It is noteworthy that most of the cultivars identified as offspring of the parental pair ‘Lechín de Granada’ × ‘Gordal Sevillana’ belong to gene pool C and share the dominant E1.1 chlorotype, corroborating the bottleneck of diversity described for Western Mediterranean in other studies [5, 21, 22, 24]. The high number of offspring attributed to this pair may reflect both the long-term coexistence of these founders and their high efficiency as crossing pair [28, 49]. Since ‘Lechín de Granada’ has the E1.1 chlorotype and ‘Gordal Sevillana’ the E1.2, it implies that the latter acted as the male progenitor [22]. This fact could be explained by a high pollinic viability of ‘Gordal Sevillana’, and an optimal phenological overlapping between these two founder cultivars. Another possible explanation could be the easier dispersal of seeds from ‘Lechín de Granada’ than from ‘Gordal Sevillana’, whose smaller fruit size allows better ingestion by bird species known to act as olive seed dispersers.

### Hybridization between local and introduced germplasm

The diverse origin of olive cultivars can also be observed at the scale of individual countries. In the case of Spain, which provided the best-represented germplasm in our study, the cultivars mainly grown in northern and north-eastern regions presented a broader diversity of origins respect to those from the South, which is in accordance with results obtained previously [11]. Many cultivars of these regions were traced back to diverse foreign ancestors or their first-degree relatives, resulting in a much more diversified network of relationships than in the south of the peninsula. ‘Gordal Sevillana’, ‘Toffehi Tataouine’, and ‘Safrawi’ were found to be a progenitor in a significant number of trios of Northern and North-eastern Spain. Interestingly, ‘Lechín de Granada’ was never inferred as a putative parent in cultivars of these regions. This suggests an influx of cultivars linked to different historical periods and different civilizations with dominium in northern or southern coast of the Mediterranean. On the other hand, the autochthonous material also seems to have played an important role in this part of the Western Mediterranean. The high level of admixture observed in this area, together with the genetic similarity to local wild forms, led some authors to suggest an autochthonous influence in some cultivars [50–53]. A cultivar that clearly exemplifies this admixture is ‘Farga’. Although ‘Farga’ harbours the western E3.1 chlorotype, its nuclear genome is more similar to cultivars carrying the eastern E1 lineage, suggesting a cytoplasmic capture through backcrossing with cultivars originating from the Eastern Mediterranean [50, 53]. This can be better understood if we analyse the putative progenitors inferred for this cultivar in our study: ‘Patrón de Cabús’ and ‘Safrawi’. There are two clues that suggest that ‘Patrón de Cabús’ may represent autochthonous material. The first is that it was assigned to gene pool B, as were other cultivars known to be closely related to wild forms, such as ‘Dokkar’ [53, 54]. The second clue comes from its name: since “Patrón” means rootstock in Spanish, it may refer to local wild material that was grafted in the past. In contrast, ‘Safrawi’ seems to be a cultivar introduced from the East by ancient civilizations, as mentioned above and in other studies [10, 24, 55]. Together, these findings support the hypothesis of hybridization between local and introduced germplasm and highlight ‘Farga’ as a likely outcome of such genetic exchange.

Furthermore, ‘Farga’ has been observed to be linked in the single parent-offspring duo to other 19 local cultivars of Northeastern Spain, suggesting that it acted as a focus of introgression and local diversification. In this context, it is worth noting that cytoplasmic male sterility (CMS) has been described in olive and is associated with the E3 chlorotype [56]. Andro-sterility within the ‘Farga’ group had already been reported by Rojas-Gómez et al., who proposed a potential founder effect [49]. This observation is consistent with our results, since ‘Farga’ carries the E3.1 chlorotype and several of its inferred descendants exhibit andro-sterility, such as ‘Carrasco’, ‘Cuquello de la Jana’, and ‘Rufina’. The co-occurrence of andro-sterility and the E3.1 haplotype therefore suggests that CMS could have been transmitted through the maternal line of ‘Farga’, further reinforcing its role as a cytoplasmic donor and as a centre of diversification in the Northeastern Iberian Peninsula.

### Practical implications for breeding and germplasm management

Apart from shedding light on the diversification history of olive, the pedigree information inferred in this study may provide substantial value for breeding programs and germplasm management. In particular, we combined paternity results with information on stigmatic compatibility groups to deduce mating behaviours, which reinforced the robustness of trios inferred with incomplete information and expanded the knowledge already available on compatibility groups [57]. This approach can improve the selection of inter-compatible genitors in future breeding programs. In this context, our analysis revealed the compatibility groups of cultivars of potential interest for modern high-density systems, such as ‘Arróniz’, ‘Rotondella di Melfi’, and ‘Zeitoun Boubezzoula’. These cultivars could represent promising alternatives to the widely used ‘Arbequina’, contributing to the diversification of intensive olive cultivation and helping to reduce risks associated with a narrow genetic base. Also, pedigree information has resulted useful for knowing the origin of progenies obtained from open pollination, or progenies received in exchanges of plant material between collections but with incomplete or lost passport information. Thirdly, knowledge of pedigrees is also useful for assembling balanced sets of progenitors with respect to relatedness. While inbreeding can be deliberately used to fix desirable traits, the extensive use of closely related progenitors may lead to increased genetic uniformity, which can limit adaptive potential and increase vulnerability to novel biotic and abiotic stresses [58]. Although inbreeding depression has not been documented in olive so far, it has been widely reported in other woody crops, where it leads to reduction in vigour, flower number and fruit set, increase in fruit abortion, lower seed germination and seedling survival, abnormal growth, and a loss of disease resistance [59]. Finally, the construction of a pedigree atlas may also have additional practical applications. For example, it can help optimize whole-genome sequencing by selecting key founders as reference points to infer missing genetic data in other cultivars [17]. Also, pedigree information can support haplotype phasing and phasing validation [60], and help distinguish identity-by-state (IBS) from identity-by-descent (IBD), particularly when working with high informative markers [61, 62]. In this context, pedigree-based and IBD-informed frameworks can also support the design of downstream analyses such as quantitative trait locus (QTL) mapping, although these applications would benefit from higher-density marker datasets [63]. In addition, pedigree information can be exploited for the description and valorisation of cultivars, serving as a resource for storytelling in cultivar marketing and providing added value, as demonstrated in other woody crops [15, 17, 19, 20].

Finally, paternity analysis should be seen as a systematic task that should be continuously updated as new cultivars are genotyped and included in the reference databases. In the case of new cultivars obtained by breeding, inferred parentage relationships can provide an opportunity for validation and quality control. For other cultivars lacking genotyping characterization and documented pedigrees, their progressive incorporation may help to refine or revise the inferred relationships. The approach used here is based on a set of 96 informative EST-SNPs [10] with intermediate allele frequencies, high polymorphism, and a very low proportion of missing data (Table S2). A substantial number of studies have demonstrated that SNP markers are as suitable for parentage analysis as classical markers, such as SSRs, and that a relatively small number (but well distributed) of SNPs, from 60 to 200, with MAF values > 0.20, are suitable for conservative pedigree inference under strict criteria [14]. In this context, our results support the suitability of this set of 96 EST-SNPs by means several independent lines of evidence. First, many of these inferences agree with those reported in previous studies using diverse sets of SSRs [21–23] or a larger set of SNPs [24]. Second, the retained robust trios were fully consistent with independent biological information, including chloroplast haplotypes, stigmatic compatibility groups, and andro-sterility. Third, the robustness of the approach was further supported by sensitivity analyses that confirmed the conservative design of the methodology, prioritizing specificity and minimizing the risk of false positive parent–parent–offspring relationships, even when one or both true parents are absent from the dataset (Table S6).

Thus, although pedigree resolution could be further improved through the use of whole-genome sequencing or higher-density SNP arrays, there is an inherent trade-off between marker density and the number of cultivars that can be feasibly genotyped. For large-scale applications such as germplasm banks and breeding platforms, extensive sampling of cultivars is critical to ensure that major founders and true parental relationships are represented in the reference dataset. In this context, the cost-effective and standardisable nature of the current method enables the genotyping of a large number of accessions under a conservative inference framework, making it particularly suitable for systematic, continuously updated, pedigree analyses. Higher-density marker approaches remain highly valuable for fine-scale resolution and targeted studies, and can be naturally integrated with pedigree inference frameworks derived from broader, lower-density datasets.

## Conclusion

The comprehensive analysis of paternity conducted here provides the first large (> 800 cultivars) olive pedigree atlas, with insights into the spread of olive cultivars along the Mediterranean Basin, but also with practical tools for modern breeding programs. The results not only confirm previously suggested genealogical links but also uncover new key founder cultivars and robust parent-offspring relationships, shedding light on the historical processes that have shaped olive cultivation. Additionally, the pedigree atlas offers actionable information for breeding and germplasm management: it enables the identification of inter-compatible parents, the reconstruction of previously unknown pedigrees, and the design of balanced genitor sets to ensure genetic diversity while minimizing the risks of inbreeding. The methodological approach used in this study demonstrates the effectiveness of a practical set of SNP markers for a rapid and a continuous pedigree analysis, which can be widely applied in germplasm management and breeding programs. This study establishes a strong basis for future research and advancements in olive breeding programs, emphasizing the need for continuous updates to the olive pedigree atlas as new cultivars are discovered and characterized.

## Supplementary Information

Supplementary Material 1.

Supplementary Material 2.

## Data Availability

The molecular marker dataset generated and analysed during the current study is not yet publicly available, as it is currently undergoing preparation and curation for deposition in the institutional repository of IFAPA under a Creative Commons Attribution (CC BY 4.0) licence. Upon completion of the repository submission process, the dataset will be made openly available and citable via a persistent identifier. In the interim, the data supporting the findings of this study are available from the corresponding author upon reasonable request.
