# Supplementary material for: An olive parentage atlas: founder cultivars, regional diversification, and implications for breeding programs
Source: BMC Plant Biol. 2026 Mar 11;26:706. doi: 10.1186/s12870-026-08504-y (PMC13088390; doi:10.1186/s12870-026-08504-y)

Figure supplementary 1: Population structure of the 853 individuals included in the study obtained from the 'LEA' admixture analysis.

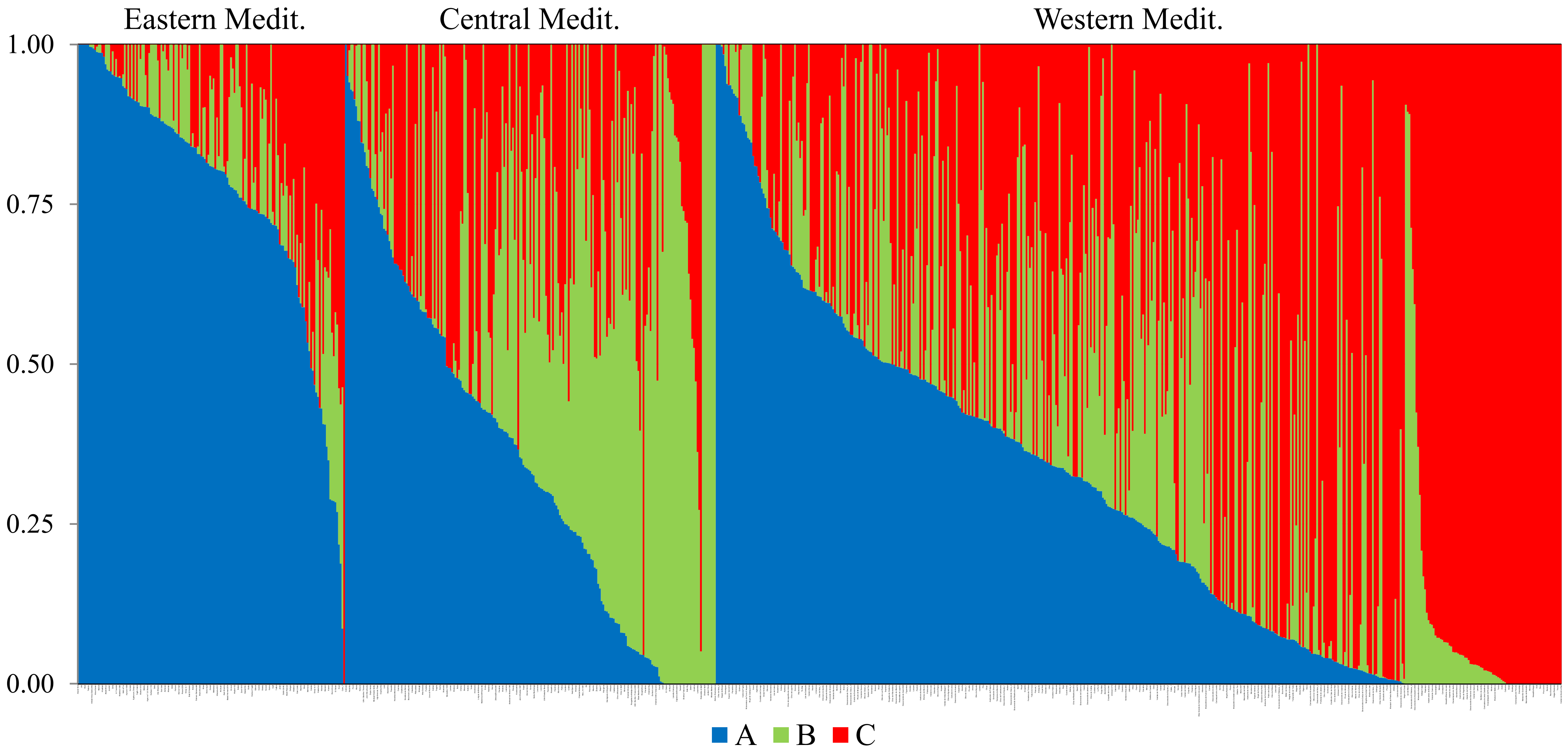

Figure supplementary 2: Distribution of trio LOD scores (LODpp) across successive filtering categories in pedigree inference

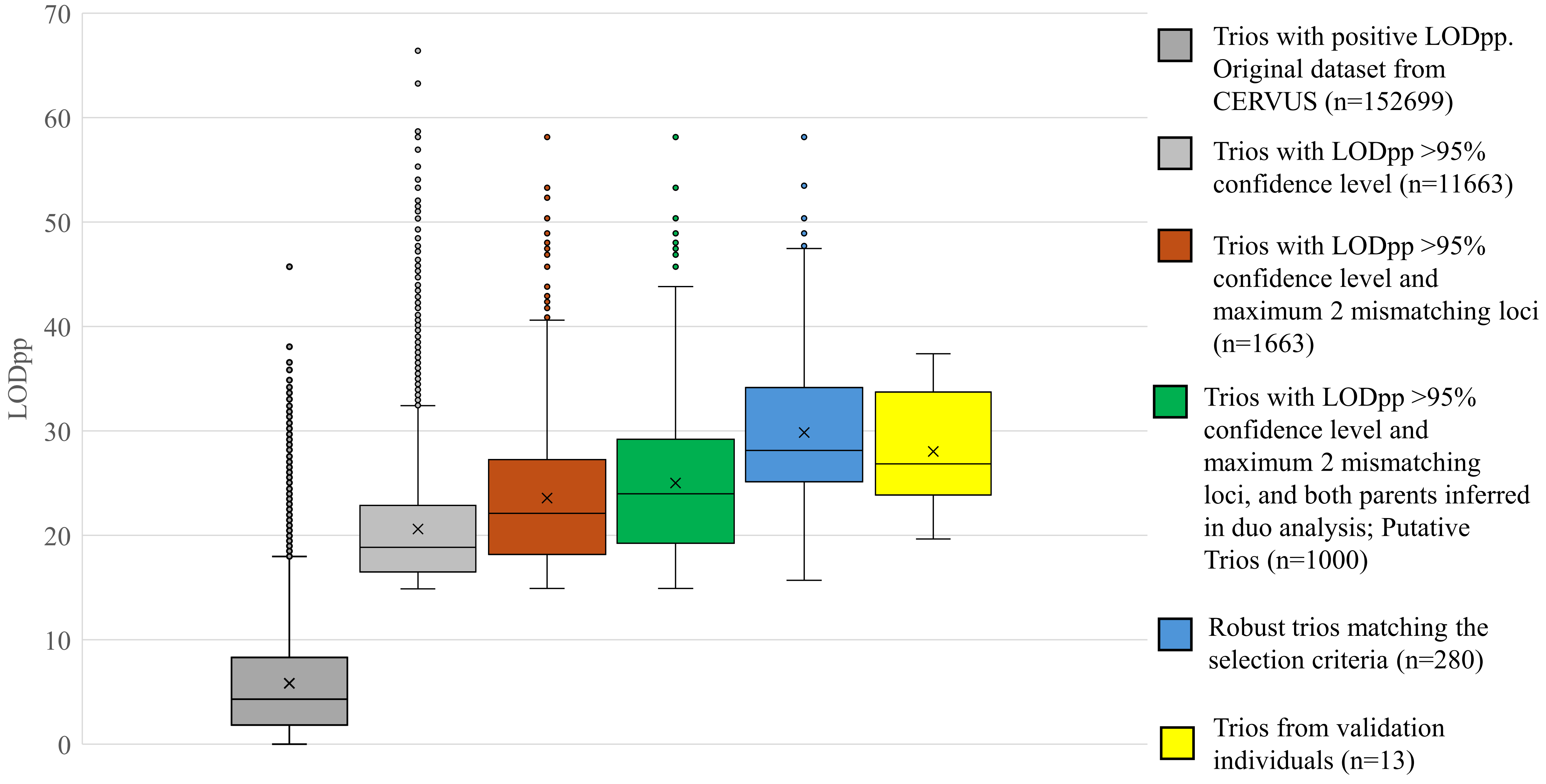

Supplement: Supplementary file 1 — Supplementary Material 1. [file 12870_2026_8504_MOESM1_ESM.pdf]
